# A flexible ultra-highly sensitive capacitive pressure sensor for basketball motion monitoring

**DOI:** 10.1186/s11671-023-03783-y

**Published:** 2023-02-17

**Authors:** Huijie Gao, Tiangeng Chen

**Affiliations:** 1 Zhuhai Sports Department, Guangdong Province, Beijing Institute of Technology, Zhuhai, 519000 China; 2 Sports Department, Capital University of Economics and Business, Beijing, 100070 China

**Keywords:** Flexible capacitive pressure sensor, Sensitivity, Dielectric layer, Basketball Motion monitoring

## Abstract

Recently, flexible sensors with high sensitivity have been applied in wearable sports sensing field. Here, we reported a flexible and sensitive capacitive pressure sensor based on nylon textile and polyvinylidene fluoride (PVDF) dielectric film. From the experimental results, the sensor has an extremely high sensitivity of 33.5 kPa^−1^, a low detection limit of 0.84 Pa, a quick response time of 27 ms. Moreover, the pressure sensor shows excellent reliability under over 100,000 working cycles. With their superior overall performance, capacitive sensors have effectively proved their enormous potential for basketball motion monitoring. This research will promote the development of wearable sports sensors.

## Introduction

With the rising need for fast-growing artificial intelligence and the Internet of Things (IoT), flexible pressure sensors, one of the critical sensing components, hold promise in medical diagnostics, medical monitoring, and wearable electronics for humans [1, 2]. It is worth noting that wearable sports training equipment based on flexible pressure sensors has received widespread attention [3]. Flexible pressure sensors generally convert applied force to a recognized electrical signal or other responsive output signal to discriminate between external stimuli [4, 5]. The flexible pressure sensor can sense various posture information of human motion [6]. Sensitivity, response time, the limit of detection (LOD), and operational stability are often used to assess the performance of pressure sensors [6–9].

Also, piezoresistive sensors, capacitive sensors, piezoelectric sensors, and triboelectric sensors are the four main types of sensors based on their detecting methods [10–12]. Flexible capacitive pressure sensors (FCPSs) have been extensively investigated because of its high sensitivity, short response time, and minimal hysteresis [13, 14]. The FCPS device may be embedded in a variety of uneven surfaces, including touchscreens, flexible displays, and even human skin [15]. The FCPS devices are made of two parallel flexible conductive planes (upper and lower electrodes) separated by a dielectric layer such as polyvinylidene fluoride (PVDF) or methacrylate [16–18]. To increase the sensitivity of FCPS, different delicate structures such as wavy, columnar, pyramidal, and micro-convex are employed as electrodes or dielectric layers [19]. For instance, Park’s group employed a porous Ecoflex dielectric layer to create a sensor with a sensitivity of 0.6 kPa^−1^ [20]. The Guo’s group achieved the sensor’s high sensitivity of 1.54 kPa^−1^ by employing microstructured rose petals and leaves as the sensor’s dielectric layer [20]. Besides, nylon mesh is a low-cost dielectric layer that may be used to increase application sensitivity (0.33 kPa^−1^, two-step replication of lotus leaves on PMDS surface as the electrode, and 0.815 kPa^−1^ sensitivity of polystyrene microsphere as a dielectric layer) [21]. However, the sensitivity of these sensors still cannot meet the needs. Therefore, the development of FCPS with high sensitivity and excellent working stability still has a long way to go [22, 23].

In this work, we reported a flexible and sensitive capacitive pressure sensor based on nylon textile and polyvinylidene fluoride (PVDF) dielectric film. As we all known, the PVDF film is a high dielectric material with good flexibility and biocompatibility; therefore, it is widely used in the field of human motion sensing. And the PVDF layer was manufactured using a simple spin-coating procedure. From the experimental results, the sensor has an extremely high sensitivity of 33.5 kPa^−1^, a low detection limit of 0.84 Pa, a quick response time of 27 ms. The sensor still maintains good stability after 100,000 cycles of continuous operation. According to results, the NSR of pressure sensor can reach 3 dB. Besides, the pressure sensor can monitor the breath, pulse, and elbow movement posture in basketball. Furthermore, the pressure sensor can also be used as a touch sensor to judge the hardness of the object, which promote its application in IoT sensor nodes.

## Experimental

### Materials

The nylon textile and conductive copper foil were purchased from the mall. Sinopharm Chemical Reagent Co., Ltd., sells sulfuric acid (H_2_SO_4_, 98%), N-methyl pyrrolidone (NMP, AR), hydrogen peroxide (H_2_O_2_, 30%), and ethanol (C_2_H_5_OH, 98%). All investigations were conducted using ultrapure water (> 18 MΩ·cm^−1^).

### Preparation of dielectric film

A PVDF/NMP solution (PVDF: NMP = 1:10, w/w) is made by dissolving the desired quantity of PVDF powder in the NMP solvent and then spinning evenly at 600 rpm for 10 s on a clean glass substrate coated with spin. The PVDF film (40 µm) was carefully pulled away from the glass after 30 min of drying at 80 °C. Dielectric layers with different thickness were created by spinning at 400 rpm for 10 s, respectively.

### Fabrication of the FCPS

FCPS was manufactured by cutting a piece of nylon textile into 1 cm × 1 cm, connecting wireway leads to a piece of external test equipment, and surface sticking one end of the cut electrode with a commercially available copper foil. Then, using the PVDF film as an intermediary layer. Then, the dielectric film is clamped in the middle with two layers of nylon film with conductive copper electrode to form the flexible pressure sensors.

## Results and discussion

**Fig. 1:**
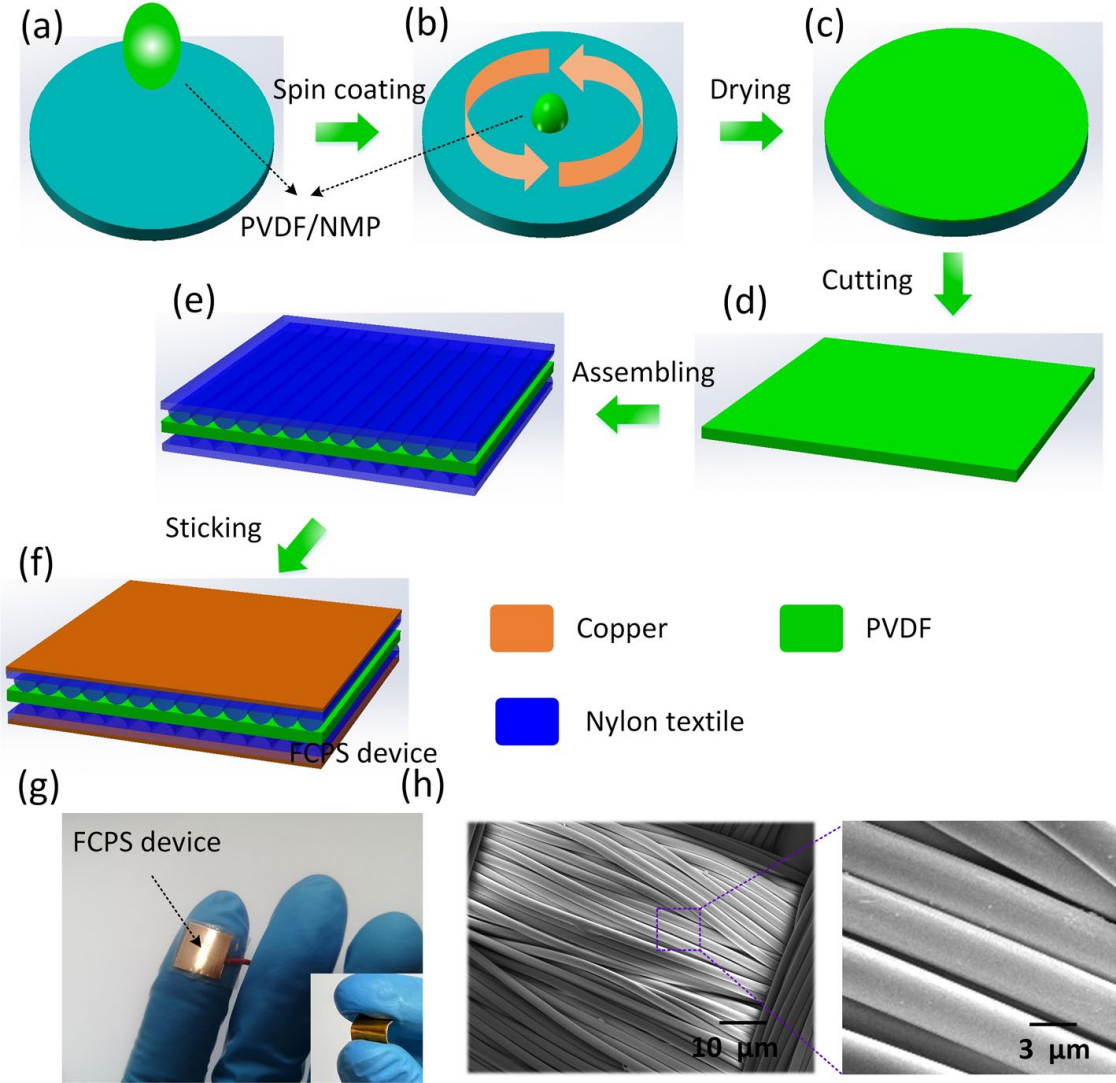
**a**–**f** The preparation process of the flexible capacitive pressure sensor. **g** The optical diagram of FCPS device, inset: the flexibility of FCPS device. **h** SEM image of the nylon textile surface

As shown in Fig. 1a-c, a piece of PVDF film was fabricated by spin coating. Then, the PVDF film was cut into 1 cm × 1 cm, as illustrated in Fig. 1d. Two pieces of nylon textile (thickness: 80 μm) wrap PVDF film in the middle, as shown in Fig. 1e. Next, a layer of copper foil (thickness: 50 μm) is pasted on the surface of nylon textile as conductive electrode, as presented in Fig. 1f. Figure 1g illustrates the picture of FCPS device. And the inset in Fig. 1g presents the flexibility of FCPS device. Figure 1h shows the SEM image of the nylon textile surface. Because nylon fiber has surface texture structure, this texture structure will deform when contacting with PVDF, resulting in the change of capacitance of the device, so as to achieve the effect of pressure sensing.

**Fig. 2:**
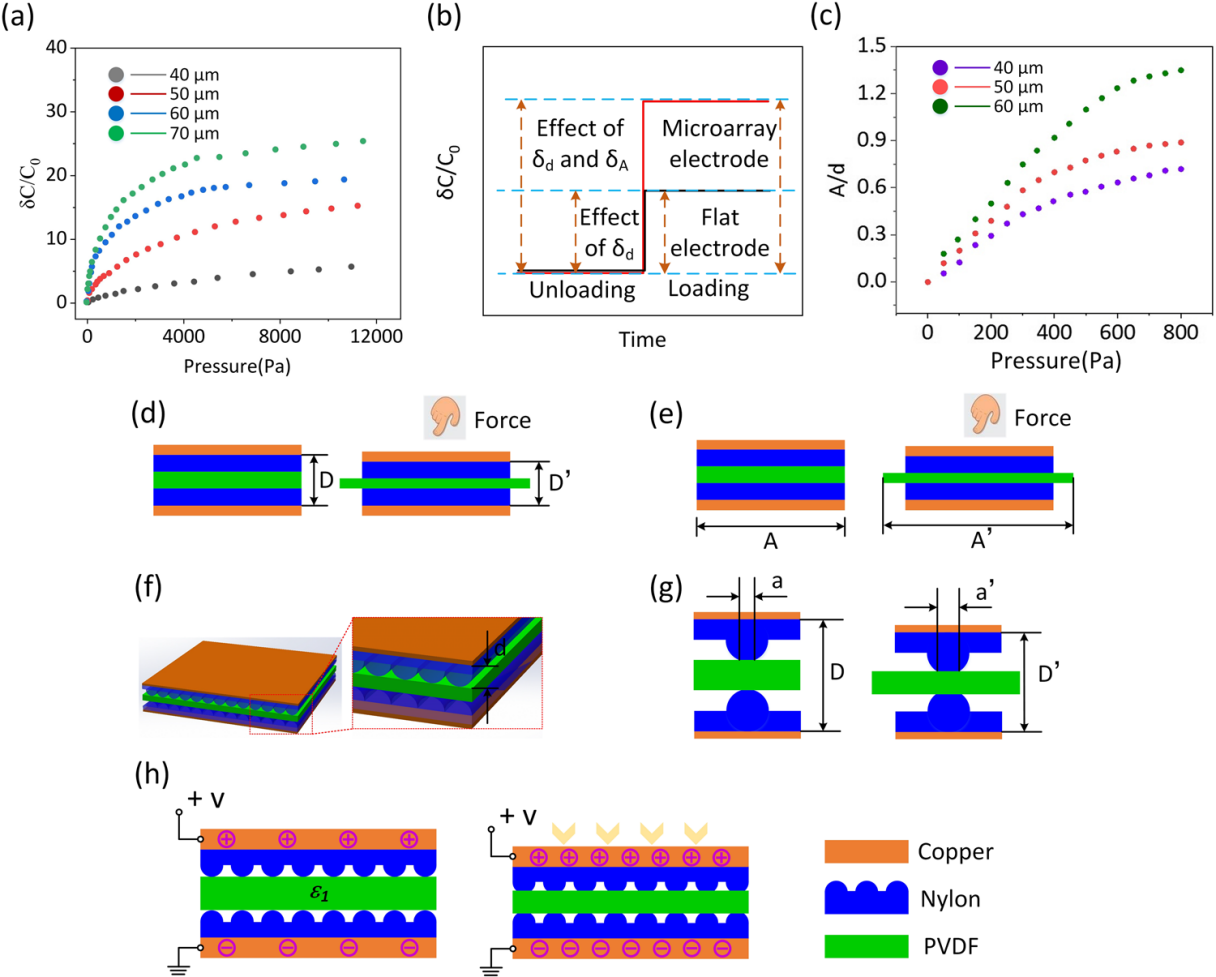
**a** The comparison of the sensitivity of FCPS device based on different thickness PVDF film. **b** The sensitivity is affected by the dielectric layer thickness. **c** The influence of A/d of pressure sensor. **d**, **e** Under the action of external force, the thickness of dielectric layer changes. **f**, **g** The changes of micromorphology under the action of external force

To evaluate the effect of dielectric layer thickness on sensing performance, the thickness of PVDF film varies from 40 to 70 µm. According to the results shown in Fig. 2a, when the thickness of PVDF film is 40 µm, FCPS has the best sensing performance. Furthermore, in the low pressure rang (0–200 Pa), the sensitivity of pressure sensor was up to 33.5 kPa^−1^, which is higher than previous work [22–25]. The synergistic impact of the altered contact area and face-to-face separation results in an exponential rise in capacitance, boosting the sensor’s sensitivity. The schematic diagram in Fig. 2b depicts the rate of change in capacitance in response to external pressure. Here, “d” and “A” denote parallel plates’ height and contact area changes, respectively. A is almost constant with regard to the pressure load applied by FCPS. The influence on capacitance changes is minimal. The d induced by the elastic dielectric layer deformation contributes to the different capacitances. In addition, we also develop the influence of the area (A) of the pressure sensor. In detail, the quantitative relationship between A/d and applied pressure is shown in Fig. 2c. In the FCPS pressure load instance, d and A both contribute to the capacitance shift, as shown in Fig. 2c, d. Thus, when pressure is applied to the FCPS, the distance between the two electrodes and the contact area changes dramatically, improving sensitivity significantly. The working mechanism of pressure sensor is shown in Fig. 2h. Due to the extremely fine texture on the surface of nylon fiber, when the pressure sensor is not under pressure, the nylon fiber layer will remain loose, which leads to the small capacitance of the pressure sensor. At the same time, when the pressure sensor is not under pressure, the PVDF film is in the maximum thickness state, which will also reduce the capacitance of the sensor. When the pressure sensor is subjected to external pressure, the loose nylon fiber will be compressed, resulting in an increase in the capacitance of the device. Furthermore, the PVDF film will also become thinner under pressure, thus increasing the capacitance of the sensor. As the capacitance of the pressure sensor changes greatly before and after being subjected to pressure, it also leads to the high sensitivity and testing range of the pressure sensor. The relevant discussion information has been added to the manuscript.

**Fig. 3:**
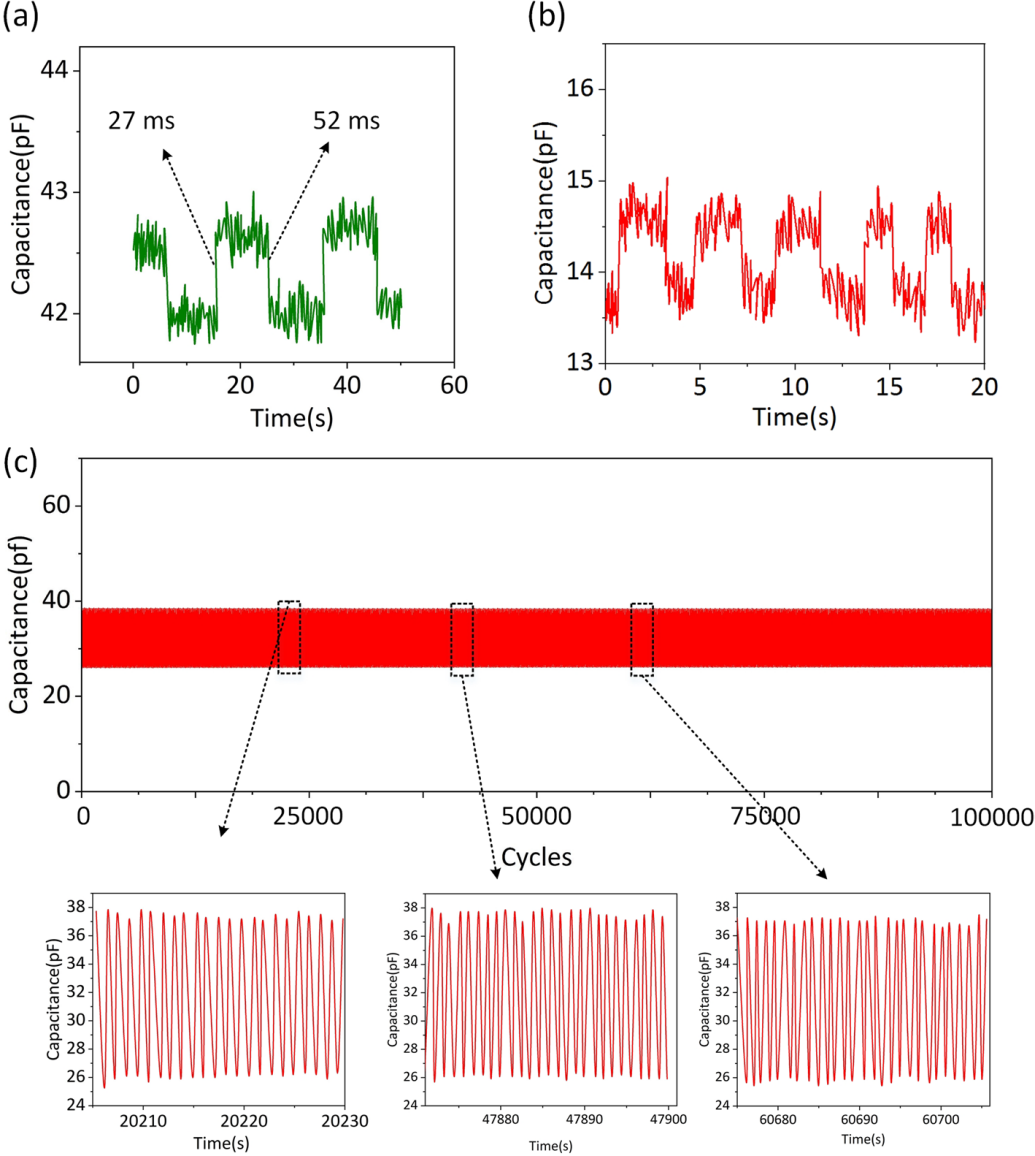
**a** The response time and relaxation time of FCPS device. **b** The output capacitance signals of FCPS device. **c** The reliability test of FCPS device

Other critical factors determined using lightweight items as external pressure are FCPS response time and detection limit. To generate a steady capacitive response signal throughout the load and unload cycles, a mica layer was put on the sensor to cover the surface. The size of the mica layer is about 1.2 cm × 1.2 cm, and the weight of the mica layer is about 95 mg. The pressure load and unload time response curves for FCPS are shown in Fig. 3a. During load and unload cycles, the harvested capacitive signals demonstrated the response time and relaxation time of 27 ms and 52 ms, respectively. As shown in Fig. 3b, a coin was put at a static pressure of 0.84 Pa on the surface of FCPS. The formula for calculating static pressure is P = mg/S. Here, m represents the mass of 32 mg of the coin, g represents the gravitational acceleration of 9.8 N/kg, and S represents the sensor’s effective area of π × 1.2 cm × 1.2 cm. The capacitance signal curve repeatedly varies when the coin is loaded and unloaded. This demonstrates that the sensor can detect and convert tiny inputs into a discernible response signal, and this sensor is better than previous work [26, 27]. Moreover, according to the characteristics of the test signal in Fig. 3a, it can be estimated that the SNR of the sensor is about 3 dB. As shown in Fig. 3c, even after 100,000 load/unload cycles, full performance is maintained, confirming FCPS repeatability and stability. The preceding findings demonstrate that the sensor as designed has considerable promise for detecting small pressures in real-world applications with high accuracy and outstanding long-term stability.

**Fig. 4:**
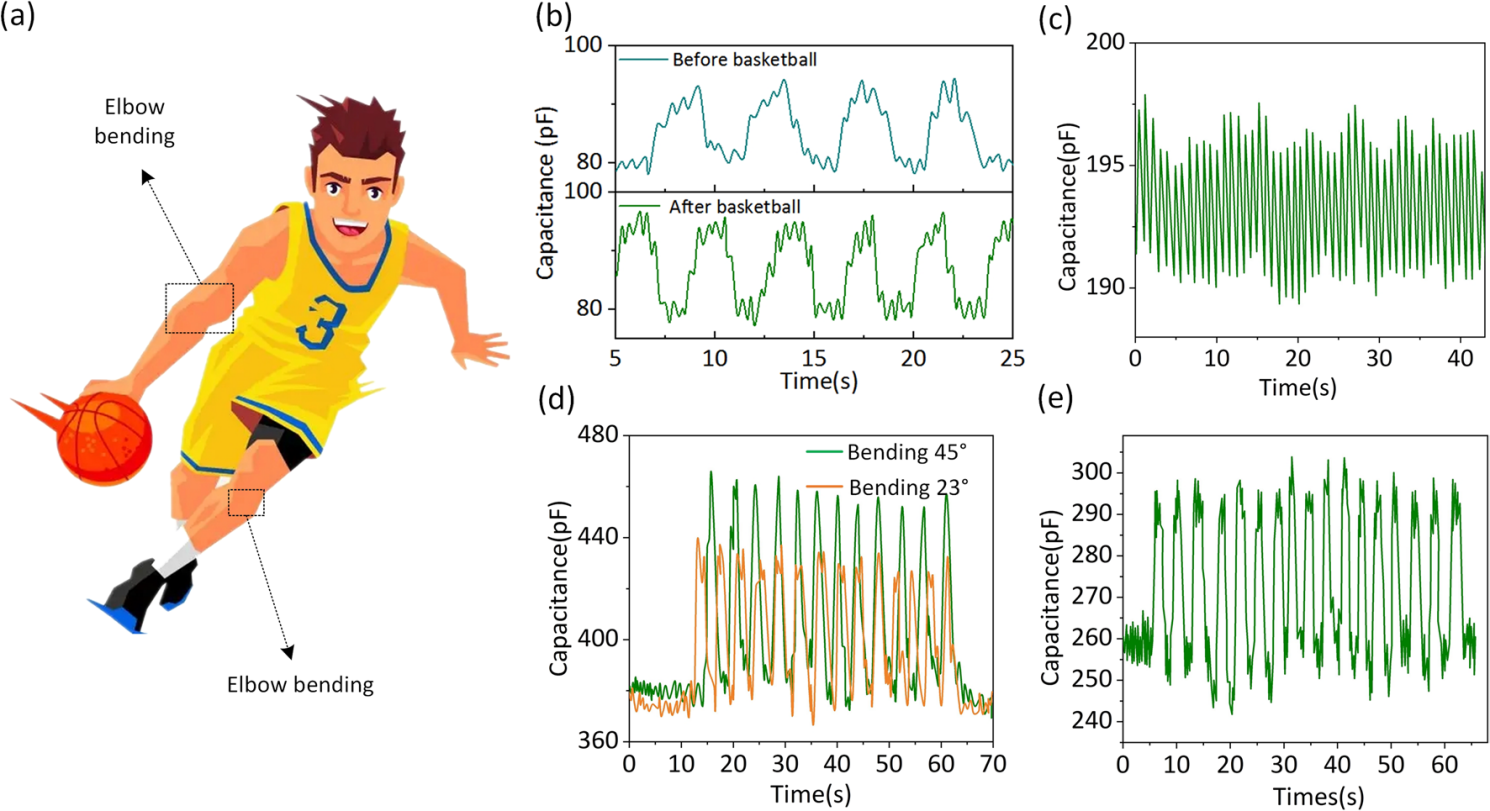
**a** Application of FCPS device in human basketball motion monitoring. **b** Output signal of FCPS device under respiratory movement before and after basketball sports. **c** The pulse monitoring signal by using FCPS. **d** The application of FCPS device in elbow bending motions with different bending angle. **e** Detection of the elbow bending movement during basketball motion

Physiological signals and bodily motions are critical for health monitoring. As shown in Fig. 4a, FCPS is utilized to detect numerous motions, such as elbow flexion in basketball motion. Specifically, the FCPS sensor is linked to several points on the human body, including the neck, wrists, arms, and legs. The measurement of respiratory rate has significant consequences for basketball motion. The respiratory signal received from the sensor to detect pressure changes caused by breath fluctuation before and after basketball sports is shown in Fig. 4b. When subjects were calm, they breathed at an average rate of 18 breaths per minute, indicating the possible use of pressure sensors in basketball motion monitoring of aberrant breathing rates. The FCPS is linked to a volunteer’s wrist to illustrate the application in pulse detection, and the findings are given in Fig. 4c. The curve graphically depicts the change in capacitance generated by the pulse and the form of a stable and repeatable pulse. Additionally, a sensor that detects the arm’s bending is linked to the joint. As shown in Fig. 4d, bending the arm about 30 and 60 degrees raises the capacitance of the sensor from 385 to 440 pF and 465 pF, respectively, and the sensor maintains a continuous bending relaxation cycle. The leg flexion signal is shown in Fig. 4e when the transducer is linked to the thigh muscle. Due to the sensor’s excellent sensitivity and stability, you may notice a consistent capacitance curve throughout the operation. These findings demonstrate the enormous potential of flexible capacitive pressure sensors as running motion monitoring devices for monitoring human basketball motion in real time.

**Fig. 5:**
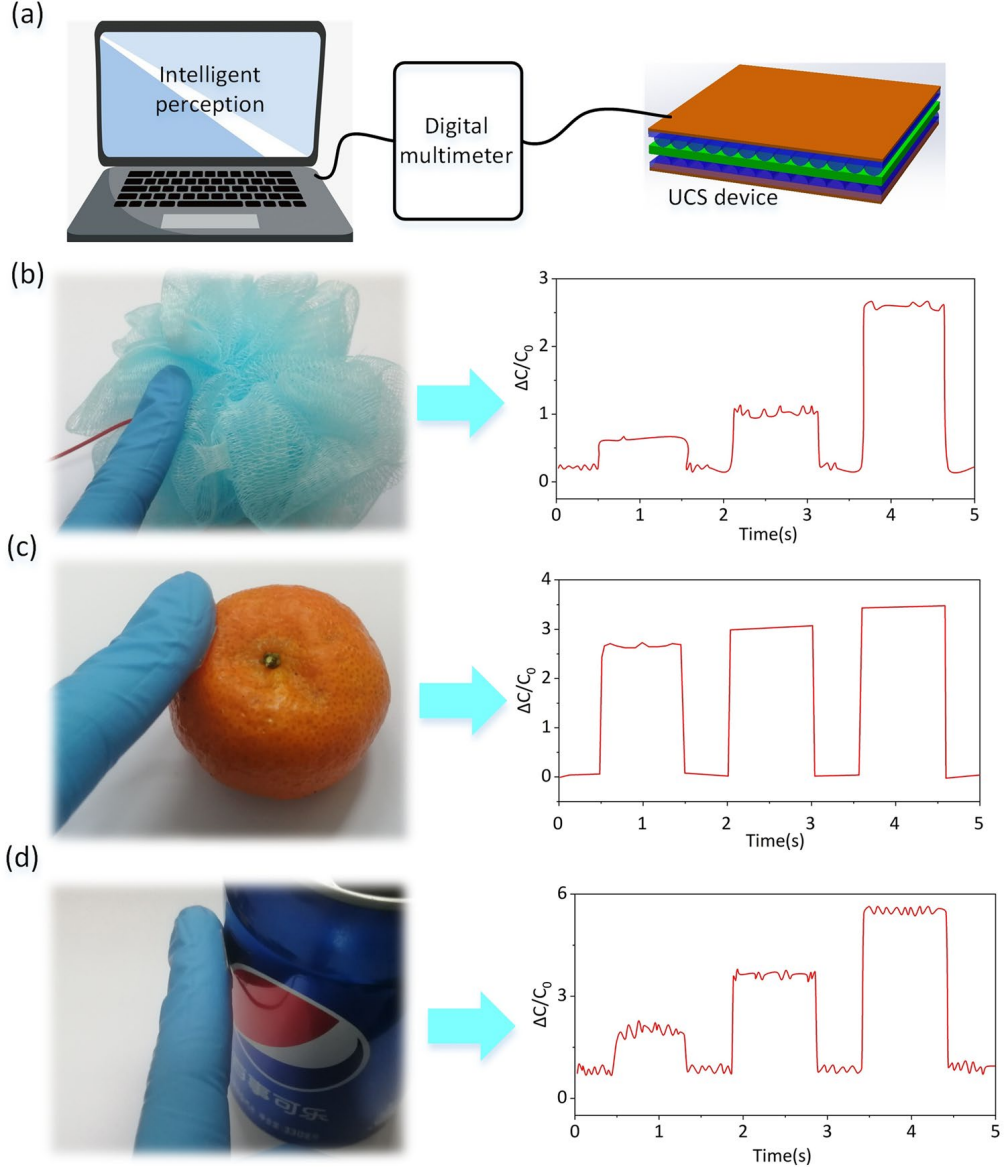
**a** Schematic diagram of touch sensing system based on FCPS device. **b-d** Perceptual signals of touching objects with different hardness, including sponges, oranges and cans

The combination of artificial intelligence and sensors determines the user’s intention in real time by tracking contextual information, such as the user’s profile, environmental conditions, and sight tracker. The combination of artificial intelligence and sensor technology is a development direction of intelligent sensor, because it improves the intelligence of sensor. To examine the FCPS-practical detecting capacity as a tactile sensor, a capacitance sensor was mounted to the surface of glove surface, as shown in Fig. 5a. An LCR tester records the capacitance signal from the sensor in real time, and LabVIEW graphically shows it on the computer screen. The electrical signal curves obtained by gripping a sponge (a soft object), a tangerine (a medium-hard item), and a can (a hard object) are shown in Fig. 5b-d. Release the grasping action for about 0.5 s, and then, reintroduce it for approximately 1 s. Repeat the above actions three times. For instance, since the sponge is light and readily distorted, the force applied to the sponge progressively rises during the grasping process. Not only is the tangerine heavy, but its surface is resistant to deformation, ensuring that the sensor signal remains constant during the gripping process. In conclusion, positive experimental findings demonstrate that capacitive sensors have great sensitivity, rapid reaction time, and excellent cycle stability, making them ideal for medical diagnostics and human–computer interaction.

## Conclusion

In summary, a low-cost and straightforward capacitive pressure sensor based on nylon textile and PVDF dielectric layer was proposed. The sensor has an extremely high sensitivity of 33.5 kPa^−1^, a low detection limit of 0.84 Pa, a quick response time of 27 ms. The sensor still maintains good stability after 100,000 cycles of continuous operation. According to results, the NSR of pressure sensor can reach 3 dB. Besides, the pressure sensor can monitor the breath, pulse, and elbow movement posture in basketball. Furthermore, the pressure sensor can also be used as a touch sensor to judge the hardness of the object, which promote its application in IoT sensor nodes.

## Data Availability

All data and materials are available without restrictions.
